# A Model of Reward- and Effort-Based Optimal Decision Making and Motor Control

**DOI:** 10.1371/journal.pcbi.1002716

**Published:** 2012-10-04

**Authors:** Lionel Rigoux, Emmanuel Guigon

**Affiliations:** 1 UPMC Univ Paris 06, UMR 7222, ISIR, Paris, France; 2 CNRS, UMR 7222, ISIR, Paris, France; University College London, United Kingdom

## Abstract

Costs (e.g. energetic expenditure) and benefits (e.g. food) are central determinants of behavior. In ecology and economics, they are combined to form a utility function which is maximized to guide choices. This principle is widely used in neuroscience as a normative model of decision and action, but current versions of this model fail to consider how decisions are actually converted into actions (i.e. the formation of trajectories). Here, we describe an approach where decision making and motor control are optimal, iterative processes derived from the maximization of the discounted, weighted difference between expected rewards and foreseeable motor efforts. The model accounts for decision making in cost/benefit situations, and detailed characteristics of control and goal tracking in realistic motor tasks. As a normative construction, the model is relevant to address the neural bases and pathological aspects of decision making and motor control.

## Author Summary

Behavior is made of decisions and actions. The decisions are based on the costs and benefits of potential actions, and the chosen actions are executed through the proper control of body segments. The corresponding processes are generally considered in separate theories of decision making and motor control, which cannot explain how the actual costs and benefits of a chosen action can be consistent with the expected costs and benefits involved at the decision stage. Here, we propose an overarching optimal model of decision and motor control based on the maximization of a mixed function of costs and benefits. The model provides a unified account of decision in cost/benefit situations (e.g. choice between small reward/low effort and large reward/high effort options), and motor control in realistic motor tasks. The model appears suitable to advance our understanding of the neural bases and pathological aspects of decision making and motor control.

## Introduction

Consider a simple living creature that needs to move in its environment to collect food for survival (foraging problem; [1]). For instance, it can have to choose between a small amount of food at a short distance and a larger amount at a longer distance [2], [3]. These two choices should not in general be equivalent as they differ by the proposed benefit (amount of food), the cost of time (temporal discounting of the benefit), and the cost of movement (energetic expenditure) [4]–[6]. To behave appropriately in its environment, our creature should be able to: 1. make decisions based on the estimated costs and benefits of actions; 2. translate selected actions into actual movements in a way which is consistent with the decision process, i.e. the criterion used *a priori* for decision should be backed up *a posteriori* by the measured costs and benefits of the selected action; 3. update its behavior at any time during the course of action as required by changes in the environment (e.g. removal or change in the position of food).

Most theories of decision making and motor control do not account for these characteristics of behavior. The main reason for this is that decision and control are essentially blind to each other in the proposed frameworks [7]. On the one hand, standard theories of decision making [8] rely on value-based processes (e.g. maximization of expected benefit), and fail to integrate the cost of physical actions into decisions [9]. On the other hand, modern theories of motor control are cast in the framework of optimal control theory, and propose to elaborate motor commands using a cost-based process (e.g. minimization of effort), irrespective of the value of actions [10], [11]. An interesting exception is the model proposed by Trommershäuser et al. [12]–[14] which casts into a Bayesian framework the observation that at least one aspect of motor control (intrinsic motor variability) is optimally integrated into decision making processes.

Here, we consider a normative approach to decision making and motor control derived from the theory of *reinforcement learning* (RL; [15]–[17]), i.e. goals are defined by spatially located time-discounted rewards, and decision making and motor control are optimal processes based on the maximization of utility, defined as the discounted difference between benefits (reward) and costs (of motor commands). The proposed mechanism concurrently provides a criterion for choice among multiple actions, and an optimal control policy for execution of the chosen action. We show that: 1. The model accounts for decision making in cost/benefit situations, and characteristics of control in realistic motor tasks; 2. Parameters that govern the model can explain the perviousness of these behaviors to motivational and task-related influences (precision, instructions, urgency). As a normative construction, the model can be considered as a prescription of what the nervous system should do [18], and is thus relevant to address and discuss the neural bases and pathological aspects of decision making and motor control. In particular, we focus on the role of dopamine (DA) whose implication in decision making, motor control and reward/effort processing has been repeatedly emphasized [2], [6], [19]–[22].

## Results

**Figure 1:**
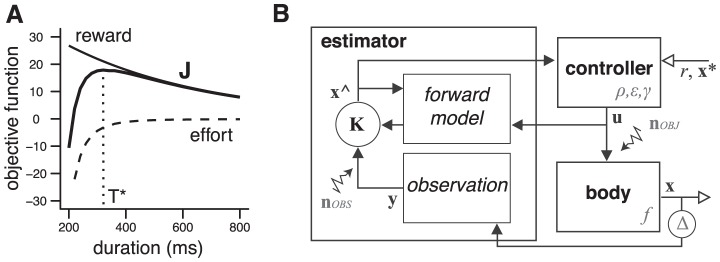
Objective function and model architecture. **A**. Objective function (*thick*) as a function of movement duration, built from the sum of a discounted reward term (*thin*) and a discounted effort term (*dashed*). Optimal duration is indicated by a vertical *dotted* line. **B**. Architecture of the infinite-horizontal optimal feedback controller. See **Text** for notations.

The proposed model is a model for decision and action. It is based on an objective function representing a trade-off between expected benefits and foreseeable costs of potential actions (Fig. 1A and Eq. 4; see **Materials and Methods**). Maximization of this function attributes a utility to each action, which can be used for a decision process, and generate a control policy to carry out the action (Eq. 6). Our goal is two-fold. First, we show that the model accounts for decision making in cost/benefit situations, and control in realistic motor tasks. Second, we show that the model makes sense from a psychological and neural standpoint. As a preliminary, we describe parameters that are central to the functioning of the model.

### Nature of the parameters

The model contains five parameters (**x** ^*^, *r*, ρ, ε, γ; Eqs. 5 and 6). Parameter **x** ^*^ specifies the location of the goal to be pursued, and acts as a classic boundary condition for a control policy. Parameter *r* is a value attached to the goal that can correspond to a reward on an objective scale (e.g. amount of food, amount of money), or to any factor that modulates the pursuit and achievement of goals (e.g. interest, attractiveness, difficulty, …). For pure motor tasks in which there is no explicit reward, we will assume that *r* corresponds to one of these factors (see **Discussion**). **x** ^*^ and *r* are parameters related to the specification of a task, and will be called *task* parameters.

For the purpose of decision and action, a reward value needs to be translated into an internal currency which measures “how much a reward is rewarding” (parameter ρ). A subject may not attribute the same value to food if he is hungry or satiated, and the same value to money if he plays Monopoly or trades at the stock exchange. *r* and ρ are redundant in the sense that only their product matters (Eq. 6), but we keep both of them because their meaning is different.

Parameter ε is a scaling factor that expresses “how much an effort is effortful”. A subject may not attribute the same value to effort if he is rested or exhausted. ρ and ε are redundant in the sense that only their ratio matters (Eq. 6), but we keep both of them because their meaning is different, and they can be regulated differently (e.g. level of wealth vs level of fatigue). In general, we consider variations in the ratio ρ/ε, that we call *vigor* factor in the following.

Parameter γ is a discount factor on reward and effort. It is both a computational parameter that is necessary to the formulation of the model, and a factor related to the process by which delayed or far away reinforcers lose value [3], [23]. Note that a decrease in γ corresponds to faster discount.

In the following, ρ, ε, and γ are called *internal* parameters, to indicate that they are not directly specified by the external environment, but correspond to a subjective valuation of concrete influences in the body and the environment. These parameters are allowed to vary to explore their role in the model. To provide a neural interpretation of the model, we tentatively relate effects of these variations to identified physiological elements.

We note that the principle of the model is independent of the values of the parameters, i.e. the decision process and the control policy are generic characteristics of the model.

### Decision making in a cost/benefit situation

**Figure 2:**
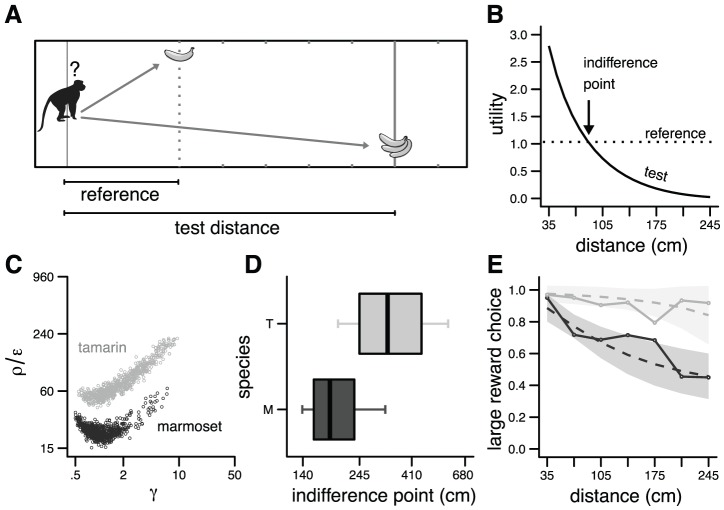
Simulation of Stevens [**3**] **.** **A**. Cost/benefit choice task between a reference option (small reward/short distance) and a test option (large reward/long distance). **B**. Utility vs distance. The dotted line indicates the utility for the reference option (*r* = 1, distance = .35 m). The solid line gives the utility for the test option (*r* = 3) for different distances (range .35–2.45 m). An arrow indicates the distance at which the preference changes. Results obtained with Object I. Parameters: ρ/ε = 1, γ = 2. **C**. Vigor and discount factors for synthetic monkeys (*black*: marmosets; *gray*: tamarins) derived from [3]. The figure was built in the following way. Mean *m* and standard deviation σ of displacement duration were obtained from Fig. 3 in [3] for each species and each amplitude. For each species, a random sample was drawn from the corresponding Gaussian distribution *N*(*m*,σ) for each amplitude, giving two durations. These two durations were used to identify a unique pair of parameters (vigor, discount). Each point corresponds to one pair. See Text for further explanation. **D**. Indifference points corresponding to the simulated monkeys shown in **C** (T = tamarin, M = marmoset). Bold bar is the median, hinges correspond to the first and third quartile (50% of the population), and whiskers to the first and ninth decile (90% of the population). **E**. Probability of choosing the large reward option according the test distance. Solid lines are the experimental data from Stevens [3]. *Dashed* lines and *shaded* areas correspond respectively to the mean and the 95% confidence interval of the decision process derived from the simulated utilities and a soft-max rule. The temperature parameter was selected for each monkey to fit empirical data.

The model provides a normative criterion for decision making when choices involve different costs and benefits. To explore this issue, we considered the simple situation depicted in Fig. 2A: a small reward at a short distance (reference distance) and a larger reward at a variable distance (test distance). Distance is used here as a way to modulate the required effort level. Simulations were run with Object I in the absence of noise. As the test distance increased, the effort to obtain the larger reward increased, and the utility decreased (Fig. 2B). Beyond a given distance (*indifference point*), the utility became smaller than the reference utility. Thus the indifference point separated two regions corresponding to a preference for the large reward/high effort and the small reward/low effort. This result corresponds to a classic observation in cost/benefit choice tasks [4], [6].

**Figure 3:**
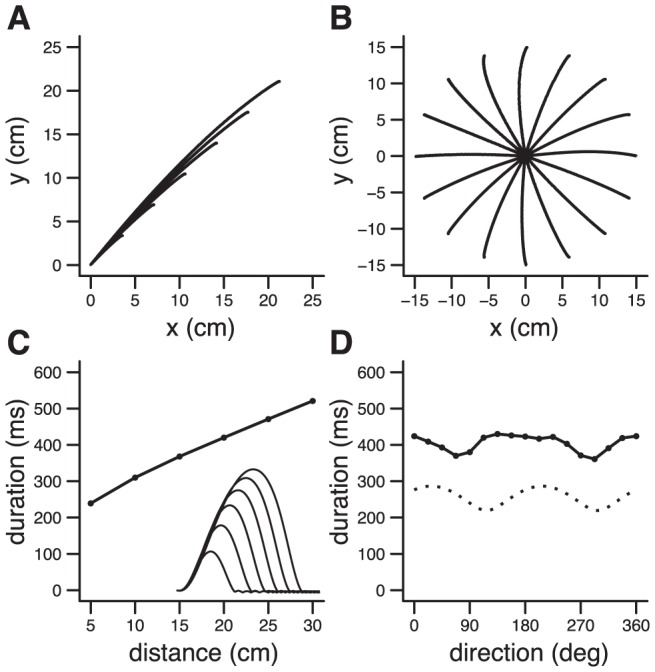
Basic characteristics of motor control. **A**. Trajectories for movements of different amplitudes (direction: 45 deg; 5, 10, 15, 20, 25, 30 cm). **B**. Trajectories for movements in different directions (10 cm). **C**. Amplitude/duration scaling law and velocity profiles (inset) for the movements in **A**. **D**. Direction/duration (*plain line*), direction/apparent inertia (*dotted line*; arbitrary unit; [31]). Results obtained with Object IIIa. Initial arm position (deg): (75,75). Parameters: *r* = 40, ρ/ε = 1/300, γ = .5, σ_SINs_ = .001, σ_SDNm_ = 1.

The model further states that the same parameters underlie both decision and movement production. To test this idea, we modeled the experiment reported by Stevens et al. [3] [referred as Stevens in the following], in which the behavior of two species of monkey (marmoset and tamarin) was assessed in the choice situation of Fig. 2A. The monkeys had to choose between one reward at 35 cm, and three rewards at 35–245 cm (distances 1 to 7). Stevens reported the choice behavior of the monkeys (Fig. 2 in Stevens) as well as the durations of chosen actions (Fig. 3 in Stevens). The modeling principle is the following. We consider that the behavior of a monkey is determined by two parameters: a vigor factor (ρ/ε) and a discount factor (γ). The question is: if we infer these parameters from the displacement duration of the monkey, can we explain its choice behavior? An important issue is the underlying determinant of amplitude/duration data (Fig. 3 in Stevens). There is strong experimental evidence for the existence of a linear relationship between distance and duration for locomotor displacements ([24]–[27]; see also [28] with fish). This observation suggests that two parameters could be sufficient to capture covariations between displacement amplitudes and durations.

For Object I, we have an analytic formula for optimal movement duration *T* ^*^(*A*,*r*,ρ/ε,γ) as a function of movement amplitude (*A*), reward (*r*), vigor (ρ/ε) and discount (γ) (see **Materials and Methods**). From Fig. 3 in Stevens, we also obtained the duration of displacement *T* (mean±s.e.m of the individual mean performances across the population) for each species in two conditions: one reward (*r_1_* = 1) located at *A_1_* = 0.35 m (marmoset: *T_1_* = .75±.061 s, tamarin: *T_1_* = .66±.047 s), and three rewards (*r_2_* = 3) at *A_2_* = 2.45 m (marmoset: *T_2_* = 1.84±.082 s, tamarin: *T_2_* = 1.32±.050 s).

We randomly drew pairs of movement duration (one for each condition) from a Gaussian distribution specified by the mean and sd ( = s.e.m×sqrt(*N*), *N* = 4) given above, thus generating for each species a set of synthetic monkeys (*n* = 100). For each sample monkey, we obtained a unique value of vigor and discount factors [two unknowns: ρ/ε and γ; two equations: *T*_1_ = *T* ^*^(*A*_1_,*r_1_*,ρ/ε,γ) and *T*_2_ = *T* ^*^(*A*_2_,*r_2_*,ρ/ε,γ)]. The corresponding parameters are shown in Fig. 2C. The two synthetic species were clearly associated with distinct regions of the parameter space, the marmosets being more sensitive to effort than the tamarins. It should be noted that Fig. 2C does not mean that there exists a redundancy between the two parameters: in fact, each point of the clouds corresponds to a different displacement behavior, i.e. different distance/duration relationships. The correlation between the parameters suggests a potential lack of specificity of the duration measurements for our method to parsimoniously characterize the populations. However, although it would be possible to tighten our predictions with more structured data (e.g. estimated parameters based on individual behavior), it is unnecessary to reveal a clear cut dissociation between the two species.

Then we computed for each monkey (i.e. for each set of parameters shown in Fig. 2C) the utility of the different options (1 reward/35 cm, 3 rewards/35–245 cm). The two sets of parameters produced different indifference points (Fig. 2D). Specifically, the majority of marmosets, in contrast with tamarins, showed an inversion in their preferences within the tested range of distances (<2.45 m).

To determine the choice behavior of the monkeys from option utilities, we calculated the probability to choose the large reward at the different distances vs the small reward at the shortest distance using a softmax rule
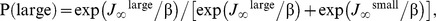
where *J*_∞_^large^ and *J*_∞_^small^ are the utilities for the large reward and small reward options, respectively, and β a temperature parameter which represents the degree of randomness of the action selection. It should be noted that the softmax transform is not a part of the model, but a way to translate utilities into choice proportions, using the natural principle that different option utilities should lead to a proportion near 1 (or 0), and equal option utilities to a proportion of 0.5. The parameter β, which had no qualitative effect on the predicted preferences, was selected for each monkey to fit the data from Stevens. The model quantitatively reproduced the empirical results in the decision task for the two monkey species (Fig. 2E). Some outliers exhibited a less characteristic behavior (whiskers in Fig. 2D) due to some imprecisions in our estimation. However, these marginal profiles were very scarce, and did not undermine our general results (see confidence interval; Fig. 2E).

To assess more precisely the ability of the model to predict the choices, we performed a detailed analysis over the two sets of simulated utilities (not over choices, to rule out any confound induced by β). We found that distance to the large reward modulated the utility of the large reward for both species, and that: 1. for tamarins, the large reward option had a larger utility than the small reward option for all distances; 2. for marmosets, the large reward option had a larger utility than the small reward option only for test distances strictly smaller than 210 cm. These results exactly parallel the effects found by Stevens, and show that the model can quantitatively predict the inversion of preferences of the different species. This further supports the hypothesis that the same process governs decision making and action in a cost/benefit choice situation.

### Control in realistic motor tasks

**Figure 4:**
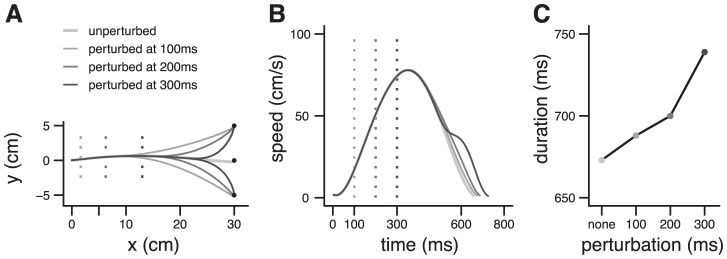
Simulation of Liu and Todorov [**29**] **.** **A**. Simulated trajectories for reaching movements toward a target which jumps unexpectedly up or down, 100 ms, 200 ms or 300 ms after movement onset. **B**. Corresponding velocity profiles. **C**. Arrival time as a function of the timing of the perturbation. Results obtained with Object IIIa. Initial arm position (deg): (15,120). Same parameters as in Fig. 3.

**Figure 5:**
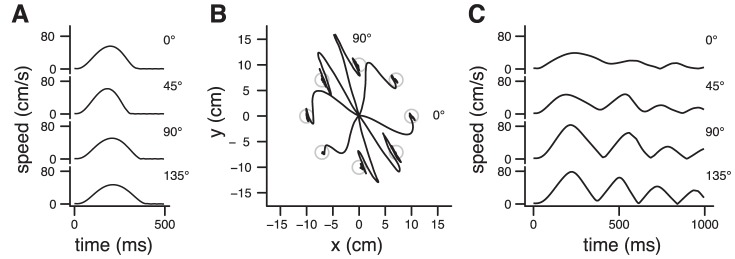
Simulation of Shadmehr and Mussa-Ivaldi [**32**]. **A**. Velocity profiles for unperturbed movements in four directions. **B**. Hand trajectories during exposure to a velocity-dependent force field. **C**. Velocity profiles for perturbed movements in four directions (data from **B**). Results obtained with Object IIIb. Initial arm position (deg): (15,100). Same parameters as in Fig. 3.

The model reproduced basic characteristics of motor behavior, as expected from the close relationship with previous optimal control models [10], [11], [29], [30]. Simulations were run with Object IIIa (two-joint planar arm) in the absence of noise. The internal parameters (ρ/ε and γ) were chosen to obtain a range of velocities compatible with observations on arm movements, and were kept constant for simulations of motor control task (Figs. 3, 4, 5). Their values had no qualitative influence on the reported results. Movements of different amplitudes (Fig. 3A) and in different directions (Fig. 3B) were considered. Simulated trajectories were straight (Fig. 3A,B) with a bell-shaped velocity profile (Fig. 3C, inset). Movement duration emerged implicitly corresponding to the best compromise between discounted rewards and efforts. Accordingly, duration was a function of movement amplitude (amplitude/duration scaling law; Fig. 3C), and movement direction (Fig. 3D, *plain line*). In fact, the influence of direction was related the inertial anisotropy of the arm (Fig. 3D, *dotted line*). Scaling was also observed for peak velocity and peak acceleration (not shown). These results are consistent with experimental observations [31].

Unexpected events can perturb an ongoing action, and prevent a planned movement to reach its goal. Typical examples are sudden changes in target location [29] or mechanical alteration of limbs dynamics [32]. In these experiments, participants correct their movements and proceed to the goal by smoothly modifying the kinematics of their arm and the duration of the action. In the model, movement duration is not fully specified in advance, but emerges from an online feedback process concerned only by the remaining effort necessary to get a reward. We wanted to test if this property could explain motor control when movement execution requires flexibility to deal with unforeseen perturbations.

In the experiment of Liu and Todorov [29], the target location jumped unpredictably during the reach. This caused a lengthening of movement duration which increased with the time elapsed between movement onset and perturbation onset (perturbation time; Fig. 1g in [29]), and systematic modifications of trajectory (Fig. 1a in [29]) and velocity profile (Fig. 1b in [29]). We simulated this task with Object IIIa by changing the goal position (**x** ^*^) in the controller at different times (perturbation time+Δ, to account for delayed perception of the change). The parameters of the model were estimated from unperturbed trials. The model quantitatively reproduced trajectory formation (Fig. 4A; Fig. 1a in [29]), velocity profiles (Fig. 4B; Fig. 1b in [29]), and the effect of perturbation time on movement duration (Fig. 4C; Fig. 1g in [29]). Liu and Todorov [29] have proposed an optimal feedback control model to explain their results. However, in their approach, the duration of perturbed movements was not an emergent property of the model, and they used experimentally measured durations in their simulations. Later in their article, they described a different model, including a cost of time, which was potentially able to predict the duration of perturbed movements, but this model was not used to explain their initial target jump data.

In the experiment of Shadmehr and Mussa-Ivaldi [32], participants performed reaching movements using a robotic device that exerted a force on their arm, i.e. altered the dynamic of their limb and continuously deflected the arm from its intended trajectory. Initial exposure to the perturbation induced deviations from straight line trajectories with typical hook-like final corrections (Fig. 7 in [32]), and multiple peak velocity profiles (Fig. 10 in [32]). We simulated this task with Object IIIb in the presence of a velocity-dependent force field. The controller was unaware of the presence of the force field. The parameters were those used in the preceding simulations (Figs. 3 and 4), and were appropriate to fit unperturbed trials. Unperturbed velocity profiles are shown for 4 directions in Fig. 5A. From the interplay between the naïve controller and the altered arm dynamics emerged curved trajectories with typical hooks (Fig. 5B), and multi-peaked velocity profiles (Fig. 5C), which are qualitatively similar to the experimental data.

These results illustrate how a unique set of parameters, and thus a unique controller, explains both normal trajectory formation, and complex updating of motor commands and trajectories when participants face unexpected perturbations. The same mechanisms (optimality, feedback control, implicit determination of duration) underlie basic motor characteristics (scaling law), and flexible control and goal tracking in complex situations.

### Modulation of decision making and motor control

The model is governed by the vigor (ρ/ε) and discount (γ) factors that can modulate both the decision process and the control policy (Eq. 6).

**Figure 6:**
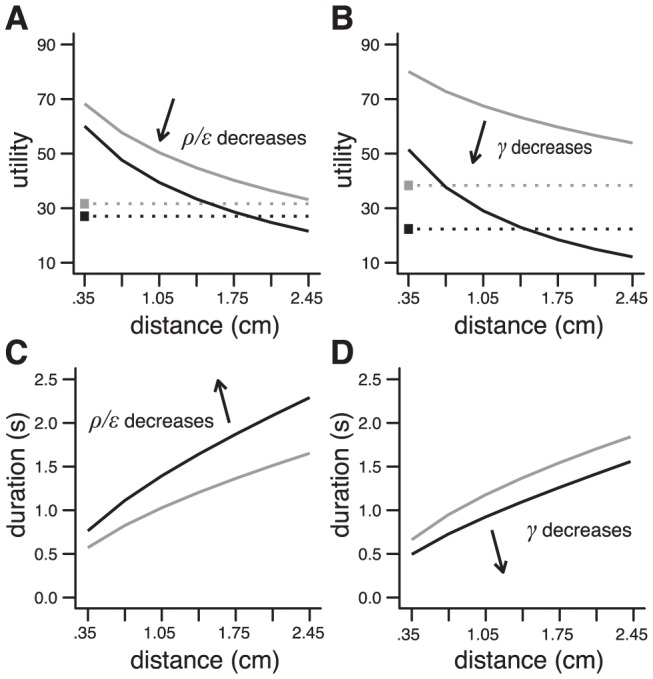
Influence of parameters. **A**. Change in the distance/utility relationship induced by a decrease in vigor: ρ/ε from 50 (*gray*) to 16 (*black*). Same experiment as in Fig. 2A. Parameters: *r* = 1, γ = 2. **B**. Same as **A** for a decrease in the value of discount factor: γ from 4 (*gray*) to 1 (*black*). Parameters: *r* = 1, ρ/ε = 50. **C**. Change in movement duration corresponding to the results in **A**. **D**. Change in movement duration corresponding to the results in **B**. Results obtained with Object I.

Decision making in a cost/benefit situation (Fig. 2A) was characterized by a threshold that delineates choice preference between small reward/low effort and large reward/high effort options (Fig. 2B). We observed a shift of the decision criterion toward the small reward/low effort option for a decreased vigor (lower ρ/ε; Fig. 6A), or a steepened discount (lower γ; Fig. 6B). Interestingly, the shift was accompanied by a decreased velocity in the former case (Fig. 6C), and an increased velocity in the latter (Fig. 6D). Note that the parameters were different from those used in Fig. 2, and were chosen here to obtain a range of velocities compatible with observations on arm movements. This choice had no influence on the results. This result is especially interesting since it reveals a dissociation between the influence of vigor and discount on decision making and motor control. The effects of vigor, but not discount, resemble the shift of decision criterion toward small reward/low effort options [2], [6], [20], and the decrease in velocity [2] observed in rat's behavior following systemic injection of dopamine receptor antagonists or DA depletion in the ventral striatum.

Motor control was characterized by scaling laws (Fig. 3C). Each factor, by its variation, defined a family of amplitude/duration scaling laws. For instance, a decrease in vigor induced an upward shift of the scaling law (Fig. 6C). Consistent with the influence of vigor described above, this result could correspond to the widely reported preservation and shift of amplitude/duration (and amplitude/velocity) scaling laws across DA manipulations and basal ganglia lesions in animals [33]–[36], and basal ganglia disorders in humans (bradykinesia; [37]–[39]). However, this interpretation is tentative as the shifts induced by vigor and discount were qualitatively similar (Fig. 6C,D; see **Discussion**).

**Figure 7:**
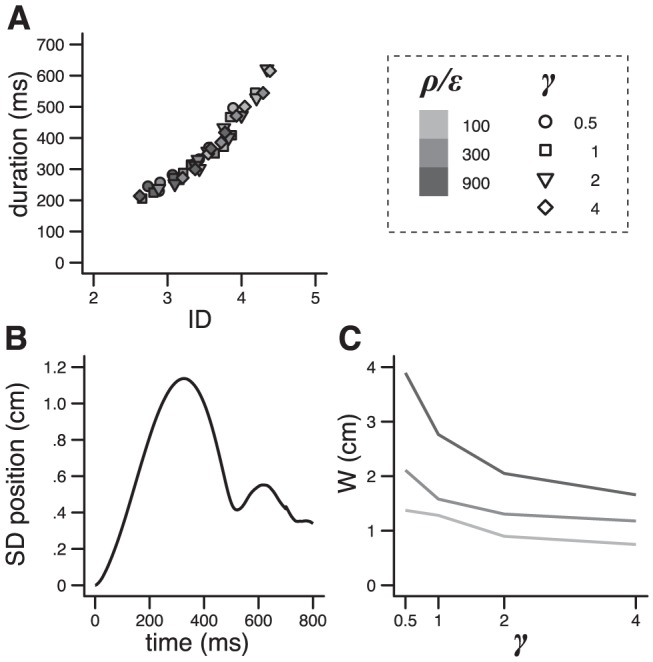
Fitts' law and variability. **A**. Duration as a function of the index of difficulty (ID) for 3 distances (10, 20 and 30 cm) and different values of vigor and discount (see legend). **B**. Typical spatiotemporal variability (s.d. of position). **C**. Endpoint variability for different values of the discount factor. Color is for the level of vigor (legend in **A**). Results obtained with Object II. Parameters: distance = 30 cm, *r* = 1, ρ/ε = 100, γ = 2, σ_SINs_ = .001, σ_SDNm_ = 1.

Along the scaling laws defined by each factor (Fig. 6C,D), amplitude, duration and variability varied in a concerted way that conformed to Fitts' law [40], [41], i.e. movement duration is a function of the index of difficulty (i.e. log_2_(2*A*/*W*), where *A* is the amplitude and *W* the endpoint variability; Fig. 7A). We note that the underlying pattern of spatiotemporal variability had two peaks, one around peak velocity and the other near the end of the movement (Fig. 7B), and is consistent with experimental observations (although the temporal profiles are usually cut before variability starts to return toward premovement levels; [29], [42], [43]). These results show that the vigor and discount factors can induce modulations of movement duration and scaling laws that might correspond to experimentally identified elements (see above and **Discussion**) while strictly obeying to a robust and ubiquitous law of motor control. Interestingly, for a given amplitude, any of these factors can act as an internal representation of a target size (Fig. 7C), i.e. it specifies a control policy that can instantaneously elaborate a movement of a given precision. It should be noted that there exist numerous models of Fitts' law in the literature [30], [44]–[46]. Our purpose here is not to propose a new model, but simply to check that Fitts' law can properly emerge from the proposed framework.

Overall, these results show that the internal parameters modulate decision making and motor control in a way that makes sense from a physiological and psychological point of view.

## Discussion

We have presented a computational framework that describes decision making and motor control as an ecological problem. The problem was cast in the framework of reinforcement learning, and the solution formulated as an optimal decision process and an optimal control policy. The resulting model successfully addressed decision making in cost/benefit situations and control in realistic motor tasks.

### Disclaimer

The proposed model is not intended to be a general theory of decision making and motor control, which may not be feasible (e.g. [47]), but a more modest theory for cost/benefit situations, i.e. specific situations in which expected benefits and foreseeable physical costs of potential actions have to be evaluated and balanced. Accordingly, the model is not concerned with classic issues of risk and uncertainty which have been thoroughly addressed in studies of Trommershäuser and colleagues [12]–[14].

### Previous models

Our model is closely related to previous works in the field of decision making and motor control. The central idea derives from optimal feedback control theory [10], and continuous time reinforcement learning [16], [17]. Several modeling studies have proposed modified versions of the optimal control approach to explain movement duration and amplitude/duration scaling laws [29], [48]–[50]. The common idea is to consider a compromise between a cost of time (which increases with movement duration), and a cost of action (which decreases with movement duration; [29], [48]–[50]). In a different framework, Niv et al. [51] proposed a compromise between a “cost of acting quickly” and a cost of “getting the reward belatedly”. In these studies, the two costs varied in opposite directions with time, and their sum had a minimum value corresponding to an optimal behavioral timing (movement duration or latency; e.g. Fig. 1B in [49]). Our model exploits the same formal idea (our Fig. 1A), but with two differences. First, the cost of time in the previous studies were chosen for specific, task-related purposes (e.g. minimize the loss of vision from image motion during a saccade in [49]; minimize the time it takes to get a target on the fovea with a saccade in [50]; see below for a further discussion on the cost of time). In our model, the cost of time derives from a general normative criterion. Second, optimization in the previous models involved only cost terms. In these approaches (e.g. [50]), a larger reward leads to a larger cost of time, thus producing a faster movements but also a lower utility, which is problematic if one wants to account for rational choices between actions. Indeed, none of these formalisms proposed to formulate motor control as a decision making problem. In our model, the reward modulates a benefit term, i.e. a larger reward leads to a larger benefit. This latter approach may be more appropriate to address cost/benefit situations in behavioral studies [52], [53], and the differential sensitivity of costs and benefits to pharmacological manipulations [52].

A series of study by Trommershäuser and colleagues [12]–[14] has explored the connection between decision making and motor control. These studies showed that human participants make optimal motor decisions (where to point in a spatial reward/penalty landscape) that take into account their intrinsic motor variability. The results suggest that at least one aspect of motor control (variability) is integrated into decision making processes (see also [54]). Our study explores a different aspect of the interaction between decision making and motor control: the influence of motor costs. In the early publications of Trommershäuser and colleagues [12], [13], a biomechanical cost was introduced, but was not actually used as it was assumed to be constant. The model described in [12], [13] is a model of decision making, which solves a spatial gain/loss trade-off at a motor planning level, but not a model of motor control as it does neither explain how movements are actually produced following a decision, nor how motor variability is estimated for a use in the decision process. Our model is primarily a model of motor control, which solves a temporal reward/effort trade-off at a motor control level, but disregards the issue of uncertainty. In this sense, our approach and that developed by Trommershäuser and colleagues [55] are complementary, and both useful to disclose the relationships between decision making and motor control.

A central and novel aspect of the model is the integration of motor control into the decision process. This idea was not exploited in previous models because movement duration was fixed [13], [56]. Our model is close to the model proposed by Dean et al. [57] (see below), as both models involve a trade-off between a time-decaying (reward) quantity and a time-increasing (accuracy in [51], minus effort in our model) quantity. However, the time-increasing quantity in [57] is derived from experimental data, and is not generated by the model, i.e. there is no normative account of the speed/accuracy relationship.

The model was described here in its simplest form. In particular, decision making was considered as a deterministic process. The scope of the model could easily be extended to address stochastic paradigms as in previous models [13], [56]. Utility needs to be replaced by mean (expected) utility or possibly mean-variance combinations [7]. Further extensions could involve subjective utilities. In fact, none of these modifications would alter the very principle of the model.

### Decision making

An analysis of behavior in terms of costs and benefits has long since been usual in behavioral ecology [1], but has only recently been exploited in the study of choice behavior in the field of neuroscience [5], [52], [58]. There is now strong evidence that not only payoff but also cost in terms of time and physical effort are integrated in the valuation of actions during a decision process [2], [6], [52], [59]. The model captures this view using an objective function in which a temporal cost is represented by a discount factor on the payoff (reward), and an effort cost by the integrated size of motor commands. The strength of this function is that it is not merely an aggregation of cost and benefit terms [50], but it has a true normative and sequential dimension [16], [17] which gives a consistent account of decision making and motor control.

A central observation in behavioral settings is that the calculation of cost involves a detailed knowledge of motor behavior [58], [59]. Experiments using parametric manipulations of costs (e.g. number of level presses) and benefits (e.g. food quantity) have shown that the choices are based on a rational ordering of actions (as measured by percentages of choice and latencies; [21]). The model also accounts for this aspect as decision is based on an exact estimation of the actual effort of tested actions derived from a complete planning process.

The study of Dean et al. [57] provides indirect evidence for the proposed decision process. In this study, subjects performed rapid arm movements to hit a rewarded target. As the reward value decayed with time (a manipulation imposed by the experimenter) and movement accuracy improved with time (natural speed/accuracy relationship), the subjects had to choose a movement duration corresponding to a trade-off between reward and accuracy (see Fig. 3 in [57]). The process described in Fig. 1A is similar, but exploits the control cost (effort) rather than the movement accuracy. This is not a critical difference since there exists an univocal relationship between effort and variability [30]. Interestingly, Dean et al. [57] observed that a majority of subjects behaved optimally in this task, i.e. chose movement durations that maximized their expected gains. These results indicate that our hypothesized optimal decision process is a feasible operation for the brain.

### Motor control

A central property of the model is motor control, i.e. the formation of trajectories for redundant biomechanical systems. This property is inherited from a close proximity with previous models based on optimal feedback control [10], [30]. A main novelty of this approach is to define a motor goal as a rewarded state rather than as a spatiotemporal constraint. Accordingly, movement duration is not a parameter, but an emerging characteristic of the interaction between a control policy, a controlled object, and unexpected events (noise, perturbations). The control policy makes no difference between a *normal* and a *perturbed* state, and always elaborates commands according to the same principle. This means that a perturbation requires neither an artificial updating of movement duration [29], nor a dual control process for early (anticipatory feedforward), and late (impedance-based) motor commands [32], [60].

### Interpreting the role of parameters

The model is governed by task and internal parameters that specify choices in cost/benefit situations, and kinematics and precision in motor tasks. These parameters have a psychological and neural dimension that we discuss below.

Parameter *r* reflects the well-documented influence of reward magnitude on decision making and intensity of action [61]–[64]. Although the observed effects are primarily mediated by physical objects (e.g. food), they can occur in the absence of reward [65], and are influenced by numerous elements. Experimental manipulations of DA transmission have been shown to bias decision making in cost/benefit situations [2], [6], [53], and alter movement intensity [2], [66]. The model offers two interpretations of these observations and of the role of DA in decision making and action, based on parameters ρ and ε (change in the perceived value of rewards or efforts). As ρ and ε have a symmetrical role, the model cannot help to decide between these interpretations. Recent studies tend to favor a relationship between effort and dopamine [19], [20], [22]. A link between ε and DA would provide a normative explanation of the strong sensitivity to response costs with preserved primary motivation for rewards following reduction of DA function [20]. Yet, the situation is probably more complex since dopamine is also involved in the valuation of reward in the absence of effort [21], [67]. Overall our results suggest that ρ and ε, through the vigor factor ρ/ε, are related to the modulation of motivational influences. Niv et al. [51] proposed the very similar idea that tonic dopamine modulates the effort to invest in a (free operant) behavior. In contrast with our work, they focused on the rate of responding irrespective of the content of the actions, i.e. motor production. The two models are grounded on the same theoretical framework, and could complementarily help to explain the dual role of dopamine in motor behavior (e.g. vigor, time discounting) and foraging behavior (e.g. rate of reward, opportunity costs).

Parameter γ has two dimensions. On the one hand, it is a *computational* parameter that is central to the infinite-horizon formulation of optimal control [17]. On the other hand, it is a psychological parameter which is widely used in behavioral ecology and economics to represent the process by which delayed reinforcers lose value [23]. What is the status of γ in the model? Two aspects need to be elucidated. First, are three parameters (ρ, ε, γ) necessary to control movement duration? Second, is γ similar to a discount factor in behavioral economics? The first question could amount to show that γ is related to nonmotivational influences. Many elements affect movement duration, such as task instructions (e.g. move accurately; [68], [69]), task difficulty [70], and task conditions (e.g. externally-triggered movements are faster than internally-triggered movements; [71]–[73]). Although it might seem clear that motivational influences are not involved in these cases, it is not easy to prove it explicitly. In this framework, the latter contrast between externally- and internally-triggered movements is especially interesting. On the one hand, this contrast is similar in normal subjects and Parkinsonian patients, both on- and off-medication [71], [72]. On the other hand, Parkinsonian patients fail to properly translate motivation into action [19], [74]. The extreme case of apathetic patients is particularly revealing as they are insensitive to incentives [74] while having “relatively spared externally-driven responses” [75]. This dichotomy is likely related to the specific implication of DA transmission in internally-generated actions [76]. Overall these results indicate that action can be modulated by influences which are independent of dopamine and motivation. The discount factor γ could mediate one of these influences.

The second question is related to the relationship between delay discounting and velocity. The study of Stevens et al. [3] is relevant to this issue. They compared the behavior of monkeys on an intertemporal choice task (a small food reward available immediately vs a delayed larger reward) and a spatial discounting choice task (a small, close reward vs a larger, more distant reward). They found that marmosets preferred larger delayed rewards in the former task, and closer, smaller rewards in the latter task. Thus their patience to wait to obtain a reward was not predictive of their will to travel farther away and for a longer time to get a larger reward. Furthermore, their travel time to the reward was not determined by their temporal discounting factor. These results indicate that decision for action is not directly governed by a discounting of time. This view is supported by neuroanatomical and neuropharmacological dissociations between effort and delay discounting in rats [2], [77]. Accordingly, the cost of time as used in the present model and in previous models [48], [49], [50], [51], seems unlike a classic temporal discounting factor, and could be specific to cost/benefit situations and motor control. This issue questions the uniqueness of time discounting across situations [50]. At odds with classical economics theories, it highlights the potential complexity and pervasiveness of the neural processes underlying computation of the cost of time [78], [79].

The model was applied to pure motor tasks in which there was no explicit reward [29], [32]. Yet, although these tasks do not apparently correspond to cost-benefit situations, there is strong experimental evidence that their execution can be modulated by cost- and benefit-related factors, e.g. loads [80], fatigue [81], task difficulty [70], attractiveness [82]. These observations suggest that pure motor tasks and reward-related motor tasks could share the same underlying representation.

### Neural architecture

The model is built on a classic control/estimation architecture (Fig. 1B), which has been thoroughly discussed in the literature [83]. There is evidence that the control process is subserved by motor cortical regions [84], [85], and the estimation process by the cerebellum [86]. A central component of the model is the translation of the task parameters into a duration, a process which involves an integration of the internal parameters to calibrate costs and benefits. As discussed above, the basal ganglia and dopamine should play a crucial role in this process. In this framework, the basal ganglia would render decision making and motor control pervious to fundamental behavioral attributes (e.g. motivation, emotion, …; [74], [87], [88]). This view is supported by studies which show that interruption of basal ganglia outputs leads to basically preserved functions [89], but deficits in behavioral modulation [74], [90].

### Testing the theory

A central proposal of the model is a common basis for decision and action. The only available data that quantitatively support this proposal are those of Stevens et al. [3], which describe both choices and displacement characteristics in a spatial discounting task (Fig. 2A). In fact, any cost/benefit decision task (e.g. T-maze; [52]) could be used to test the theory if data on displacement duration were available. As in [3], there should be a univocal relationship between displacement characteristics and choice behavior. A failure to observe this relationship would falsify the model. This would in fact correspond to a self-contradictory behavior: the costs and benefits that are estimated at the time of the decision would not be equal to those effectively encountered (during and after the movement). It should be noted that this failure would not be of the same nature as that usually reported in the field of decision making (e.g. a deviation from the laws of probabilities).

The preceding results involved locomotor patterns, but appropriate data for arm movements could be obtained using methods described in [59]. In a different domain, the model suggests that movement intensity can be modulated by nonmotivational elements, represented by the discount factor γ. One element could be urgency [71], [72]. This issue could be tested in apathetic patients, who should show a preserved sensitivity to urgency despite a loss of sensitivity to incentives [74].

## Materials and Methods

Our objective is to formulate a unified model of decision making and motor control. Classical normative approaches formalize decision making as a maximization process on a *utility function* [91], and motor control as a minimization process on a *cost function* [92]. Our proposal is to build a global *utility minus cost* function (that we call again a utility function) that could govern choices and commands in a unitary way. The central issue is time, because costs in motor control are a function of time (i.e. slower movements are less costly than faster movements), as are rewards due to a discounting effect (i.e. late rewards are less valuable than immediate ones). This means that a rational choice between two actions should involve an evaluation of their durations. However, the duration of the chosen action is only a prospective duration, valid at a given time, based on the assumption that current conditions will not change until the end of movement. The actual duration of the action can differ from this prospective duration if unexpected perturbations are encountered during the course of its execution.

We have arbitrarily chosen the notations of control theory (*J* for utility/cost function, *u* for control) rather than those of decision theory (*U* for utility function, *a* for action).

The principles of the model are first explained on a simple, deterministic example. Then the complete, stochastic version is described. The model is cast in the framework of reinforcement learning although we only exploit the optimal planning/decision processes of RL, but not the learning processes. The rationale for this choice is the following. Formally, the model corresponds to an infinite-horizon optimal control problem [93]. This jargon is typically used in economics [94], but is much less familiar in the fields of motor control and decision making, which describe similar problems in the terminology of RL [15], [16]. Furthermore, the RL framework encompasses learning processes which could explain how the proposed operations are learned by the nervous system [95], [96].

### A starting example

We consider an inertial point (controlled object) described by its mass *m* and its state **x** = (*p*,*v*) (where *p* and *v* are the position and velocity of the object; **bold** is for vectors). This object can move along a line, actuated by a force generating system (e.g. a set of muscles). The force generating system is defined by a function *h* which translates a control vector **u** into muscular force ([97]; *h* needs not be specified for the moment). This is a simplistic case to address e.g. the control of unidimensional saccades or single joint movements [10], [49]. The dynamics of the point is given by the general equation
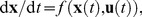
(1)corresponding to
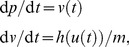
(2)in the case of a single muscle. To control this object means finding a *control policy*, i.e. a function **u**(*t*) (*t*∈[*t*_0_;*t*_f_]) that can displace the point between given states in the duration *t*_f_−*t*_0_. In the framework of the optimal control theory, the control policy is derived from the constraint to minimize a cost function
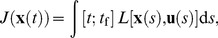
(3)for any time *t*∈[*t*_0_;*t*_f_], where *L* is a function which generally penalizes large controls (*effort*) and deviations from a goal state (*error*; see [92] for a review). This formulation is appropriate to solve the problem of motor control, i.e. the mastering of the dynamics of articulated mechanical systems [10], but does not directly apply to a foraging problem (as described in the **Introduction**) for at least two reasons. First, function *L* is not concerned with values in the environment, although this difficulty could be relieved by the addition of a value-related term. Second, and more fundamental, the objective function cannot be used to specify the duration of an action, or to attribute a value to an action independent of its duration. Thus *J* cannot be considered as a utility function for decision making among multiple actions.

An alternative approach has been elaborated as an extension of RL in continuous time and space [16]. In this case, an infinite-horizon formulation is used where the error/effort cost function is replaced by a time-discounted, reward/effort function (to be maximized in this case)
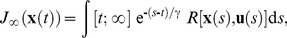
(4)where *R* is a function which weights rewarding states positively and effort negatively, and γ a time constant for discounting reward and effort. As for Eq. 3, Eq. 4 gives a recipe to find an optimal control policy **u**(*s*) for *s*∈[*t*;∞]. For clarity, we use the symbol γ for the discount parameter as usually found in RL studies [15]. Yet the range for the discount factor is [0;1] for discrete RL, and [0;+∞[ for the continuous-time formulation used here (see [16] for a correspondence between discrete and continuous RL). As in RL, a small value of γ corresponds to a large discounting effect.

We consider the case of a simple *reward minus effort* function where there is a single reward of value *r* at state **x** ^*^, i.e.
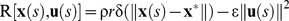
(5)where δ is the function which is 1 when **x**(*s*) = **x** ^*^, and 0 everywhere else, and ρ and ε are scaling factors for reward and effort, respectively (see **Results** for a complete description of the parameters). If the inertial point starts to move at time *t*, reaches the rewarded state at an unknown time *T*, and the reward is given for a single timestep, we can write from Eq. 4 and Eq. 5, using the fact that **u**(*s*) = 0 for *s*>*T* (the point stays indefinitely at the rewarded state)
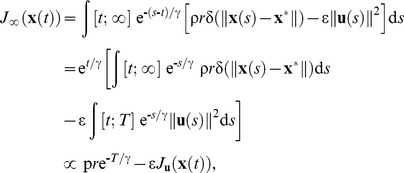
(6)


where the term ρ*r*e^−*T*/γ^ is the discounted reward (this result comes from the fact that ∫ *g*(*s*)δ(*s*)d*s* = *g*(0) for any function *g*), and *J* **_u_**(**x**(*t*)) is the motor cost
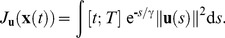
(7)We have removed the term exp(*t*/γ) which has no influence on the maximization process. This point highlights the fact that the maximization process does not depend on current time *t*. For clarity, in the following, *J*_∞_ and *J* **_u_** are considered as functions of the reward time *T*.

The purpose of Eq. 6 is, as for Eq. 3, to obtain an optimal control policy. Maximizing *J*_∞_ requires to find a time *T* and an optimal control policy **u**(*s*) for *s*∈[*t*;*T*] that provide the best compromise between the *discounted reward* (ρ*r*e^−*T*/γ^) and the *effort* (*J* **_u_**). This point is illustrated in Fig. 1A. Both the discounted reward and the effort (−*J* **_u_** is depicted) decreases with *T* (i.e. a faster movement involves more effort, but leads to a less discounted reward while a slower movement takes less effort, but incurs a larger discount), and their difference takes a maximum value at a time *T* ^*^ (*optimal duration*). For each *T*, the control policy is optimal, and is obtained by solving a classic *finite-horizon* optimal control problem with the boundary condition **x**(*T*) = **x** ^*^ ([98], [99]; see below). We note that *T* ^*^ may not exist in general, depending on the shape of the reward and effort terms (Fig. 1A). Yet, this situation was never encountered in the simulations. The search of an optimal duration can be viewed both as a decision-making process (decide what is the best movement duration *T* ^*^ if it exists), and a control process (if *T* ^*^ exists, act with the optimal control policy defined by *T* ^*^). In the following, the maximal value of *J*_∞_ (for *T* = *T* ^*^) will be called *utility*.

This description in terms of duration should not hide the fact that duration is only an intermediate quantity in the maximization of the utility function, and direct computation of choices and commands is possible without explicit calculus of duration [95], [96].

If there are multiple reward states in the environment, the utility defines a normative priority order among these states. A decision process which selects the action with the highest utility will choose the best possible cost/benefit compromise.

The proposed objective function involves two elements that are central to a decision making process: the benefit and the cost associated with a choice. A third element is uncertainty on the outcome of a choice. In the case where uncertainty can be represented by a probability (risk), this element could be integrated in the decision process without substantial modification of the model. A solution is to weight the reward value by the probability, in order to obtain an “expected value”. Another solution is to consider that temporal discounting already contains a representation of risk [100].

In summary, equations (4) and (5) are interesting for four reasons: 1. Movement duration emerges as a compromise between discounted reward and effort; 2. The objective function is a criterion for decision-making either between different movement durations, or between different courses of action if there are multiple goals in the environment; 3. The objective function subserves both decision and control, which makes them naturally consistent. The utility that governs a decision is exactly the one that is obtained following the execution of the selected action (in the absence of noise and perturbations); 4. The objective function does not depend explicitly on time, which leads to a stationary control policy [16], [17].

### General framework

For any dynamics (Eq. 1), the problem defined by Eqs. 4 and 5 is a generic infinite-horizon optimal control problem that leads, for each initial state, to an optimal movement duration and an optimal control policy (see above). This policy is also an optimal feedback control policy for each estimated state derived from an optimal state estimator [10], [99], [101], [102]. Thus the current framework is appropriate to study online movement control in the presence of noise and uncertainty. The only difference with previous approaches based on optimal feedback control [10], [99] is that movement duration is not given *a priori*, but calculated at each time to maximize an objective function.

The general control architecture is depicted in Fig. 1B. As it has been thoroughly described previously [30], [98], [99], [103], we only give here a rapid outline. The architecture contains: 1. A *controlled object* whose dynamics is described by Eq. 1, and is corrupted by noise **n**_OBJ_; 2. A *controller* defined as
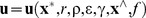
(8)which is an optimal feedback controller for Eqs. 1, 4, 5, where **x** ^∧^ is the state estimate (described below); 3. An *optimal state estimator* that combines commands and sensory feedback to obtain a state estimate **x** ^∧^ according to
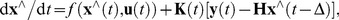
(9)where **K** is the Kalman gain matrix [constructed to provide an optimal weighting between the output of the forward model (first term in the rhs of Eq. 9), and the correction based on delayed sensory feedback (second term in the rhs of Eq. 9)], **H** the observation matrix, **y**(*t*) = **Hx**(*t*−Δ)+**n**_OBS_ the observation vector corrupted by observation noise, and Δ the time delay in sensory feedback pathways. The observed states were the position and velocity of the controlled object.

Object noise was a multiplicative (signal-dependent) noise with standard deviation σ_SDNm_, and observation noise was an additive (signal-independent) noise with standard deviation σ_SINs_ [98]. The rationale for this choice is to consider the simplest noisy environment: 1. Signal-dependent noise on object dynamics is necessary for optimal feedback control to implement a minimum intervention principle [10], [99]; 2. Signal-independent noise on observation is the simplest form of noise on sensory feedback. We note that a stochastic formulation was necessary to the specification of the state estimator even though most simulations actually did not involve noise.

### Simulations

A simulation consisted in calculating the utility (maximal value of the objective function), and the timecourse of object state and controls for a given dynamics *f*, initial state, and parameters **x** ^*^, *r*, ρ, ε, γ, σ_SINs_, σ_SDNm_, Δ. The solution was calculated iteratively at discretized times (timestep η). At each time *t*, a control policy was obtained for the current state estimate **x** ^∧^ (Eq. 8). Two types of method were necessary. First, the integral term in the rhs of Eq. 6 (Eq. 7) required to solve a finite-horizon optimal control problem. This problem was solved analytically in the linear case, and numerically in the nonlinear case (see below). Second, optimal movement duration was obtained from Eq. 6 using a golden section search method [104]. Then Eqs. 1 and 9 were integrated between *t* and *t*+η for the selected control policy and current noise levels (σ_SINs_, σ_SDNm_) to obtain **x**(*t*+η) and **x** ^∧^(*t*+η). The duration of the simulation was set empirically to be long enough to guarantee that the movement was completely unfolded. Actual movement duration (and the corresponding endpoint) was determined from the velocity profile using a threshold (3 cm/s).

Three types of object were considered, corresponding to different purposes. The rationale was to use the simplest object which is deemed sufficient for the intended demonstration. Object I was a unidimensional linear object similar to that described in the starting example. The force generating system was *h*(*u*) = *u*. This object was used for decision making in a cost/benefit situation. Object II was similar to Object I, but the force generating system was a single linear second-order filter force generator (time constant τ), i.e. the dynamics was
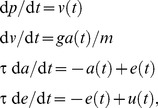
(10)where *a* and *e* are muscle activation and excitation, respectively, and g = 1 a conversion factor from activation to force. The filtering process is a minimalist analog of a muscle input/output function [105]. This object was used to study motor control in the presence of noise (relationship between amplitude, duration, and variability) [10], [30], [45]. In this case, variability was calculated as the 95% confidence interval of endpoint distribution over repeated trials (*N* = 200). Object III (IIIa and IIIb) was a classic two-joint planar arm (shoulder/elbow) actuated by two pairs of antagonist muscles. The muscles were described as nonlinear second-order filter force generators. All the details are found below. This object was used to assess characteristics of motor control in realistic motor tasks.

### Parameters

For Objects I and II, the mass *m* was arbitrarily chosen to be 1 kg (no influence on the reported results). For Object III, the biomechanical parameters are given below. Other fixed parameters were: τ = 0.04 s, Δ = 0.13 s, η = 0.001 s. Noise parameters (σ_SINs_, σ_SDNm_) were chosen to obtain an appropriate functioning of the Kalman filter, and a realistic level of variability. The remaining parameters (**x** ^*^, *r*, ρ, ε, γ) are “true” parameters that are varied to explore the model (see **Results**).

### Model of the two-joint planar arm

Object III is a two-joint (shoulder, elbow) planar arm. Its dynamics is given by
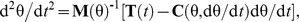
where θ = (θ_1_,θ_2_) is the vector of joint angles, **M** the inertia matrix, **C** the matrix of velocity-dependent forces, **W** an optional velocity-dependent force field matrix, and **T**(*t*) the vector of muscle torques defined by
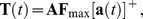
where **A** is the matrix of moment arms, **F**_max_ the matrix of maximal muscular forces, and **a** the vector of muscular activations resulting from the application of a control signal **u**(*t*) (see Eq. 10).

For each segment (1: upper arm, 2: forearm), *l* is the length, *I* the inertia, *m* the mass, and *c* the distance to center of mass from the preceding joint. Matrix **M** is
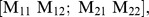
with
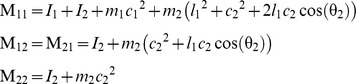
Matrix **C** is
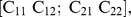
with
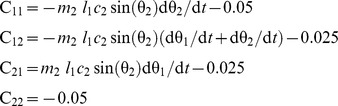
Matrix **W** is **JDJ** *^T^*, where **J** is the Jacobian matrix of the arm, and **D** (Ns/m) is
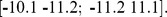
Matrix **F**_max_ (N) is diag([700;382;572;449]). Matrix **A** (m) is
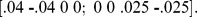



Two sets of parameter values were used in the simulations. For Object IIIa, we used the values found in [29] (in S.I.): *l*_1_ = .30, *l*_2_ = .33, *I*_1_ = .025, *I*_2_ = .045, *m*_1_ = 1.4, *m*_2_ = 1.1, *c*_1_ = .11, *c*_2_ = .16. For Object IIIb, we used the values given in [32]: *l*_1_ = .33, *l*_2_ = .34, *I*_1_ = .0141, *I*_2_ = .0188, *m*_1_ = 1.93, *m*_2_ = 1.52, *c*_1_ = .165, *c*_2_ = .19.

### Resolution of the optimal control problem

The problem is to find the sequence of control **u**(*t*) which optimizes the objective function *J_u_*(*T*) (Eq. 7), and conforms to the boundary conditions **x**(*t*_0_) = **x**_0_ and **x**(*T*) = **x** ^*^ for a given dynamic *f*. The general approach to solve this problem is based on variational calculus [106]. The first step is to construct the Hamiltonian function which combines the objective function and the dynamic thanks to the Lagrangian multipliers (or co-state) denoted by λ
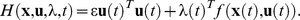
The optimal control minimizes the Hamiltonian, a property known as the Pontryagin's minimum principle given formally by
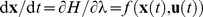
(11)

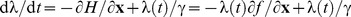
(12)

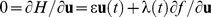
(13)
Equation (12), widely used in economics, is slightly different from what is usually used in the motor control literature because of the discounting factor in the objective function. We will thereafter consider two methods to solve this set of differential equations depending on the complexity of the dynamics.

#### Linear case

If the dynamic *f* is linear, as for Objects I and II, the system of differential equations (Eqs. 11, 12, 13) is also linear, and can be solved analytically. We rewrite the dynamics as
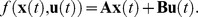
From Eq. 13, we can reformulate the optimal control **u** ^*^(*t*) as
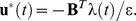
In order to find λ(*t*), we then replace **u**(*t*) by **u** ^*^(*t*) in Eqs. 11 and 12, and get
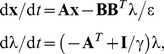
where **I** is the identity matrix. The resolution of this system gives the optimal trajectory of the state and the co-state
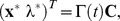
where Γ is the analytic solution to Eq. 14, and **C** can be deduced from the boundary conditions [99]. Finally, we replace λ by λ^*^ in Eq. 14 to get the value of the optimal control. From Eq. 6, we obtain an analytic version of the utility, from which we can derive the optimal duration *T* ^*^ analytically. Symbolic calculus was performed with Maxima (Maxima, a Computer Algebra System. Version 5.18.1 (2009) http://maxima.sourceforge.net/).

#### Nonlinear case

When the dynamics is nonlinear (Object III), the set of differential equations (Eqs. 11, 12, 13) cannot be solved directly. However, the minimum of the Hamiltonian (and thus the optimal control) can be found through numerical methods using a gradient descent method. The detail of the existing algorithms is outside the scope of this article, and the reader is referred to [101], and [106].
